# *N*-(4-Chloro­phen­yl)-2,2-diphenyl­acetamide

**DOI:** 10.1107/S1600536812032965

**Published:** 2012-07-25

**Authors:** Hoong-Kun Fun, Wan-Sin Loh, Prakash S Nayak, B. Narayana, B. K. Sarojini

**Affiliations:** a X-ray Crystallography Unit, School of Physics, Universiti Sains Malaysia, 11800 USM, Penang, Malaysia; b Department of Studies in Chemistry, Mangalore University, Mangalagangotri 574 199, India; c Department of Chemistry, P.A. College of Engineering, Nadupadavu, Mangalore 574 153, India

## Abstract

In the title compound, C_20_H_16_ClNO, an *S*(6) ring motif is formed *via* an intra­molecular C—H⋯O hydrogen bond. The chloro-substituted benzene ring is almost perpendicular to the benzene rings, forming dihedral angles of 87.33 (9) and 88.69 (9)°. The dihedral angle between the benzene rings is 87.17 (9)°. In the crystal, mol­ecules are linked into chains parallel to the *c* axis by inter­molecular N—H⋯O hydrogen bonds. The crystal packing also features weak C—H⋯π inter­actions involving the chloro-substituted ring.

## Related literature

For related structures, see: Fun *et al.* (2012*a* ▶,*b* ▶,*c* ▶). For hydrogen-bond motifs, see: Bernstein *et al.* (1995 ▶). For bond-length data, see: Allen *et al.* (1987 ▶). For the stability of the temperature controller used in the data collection, see: Cosier & Glazer (1986 ▶).
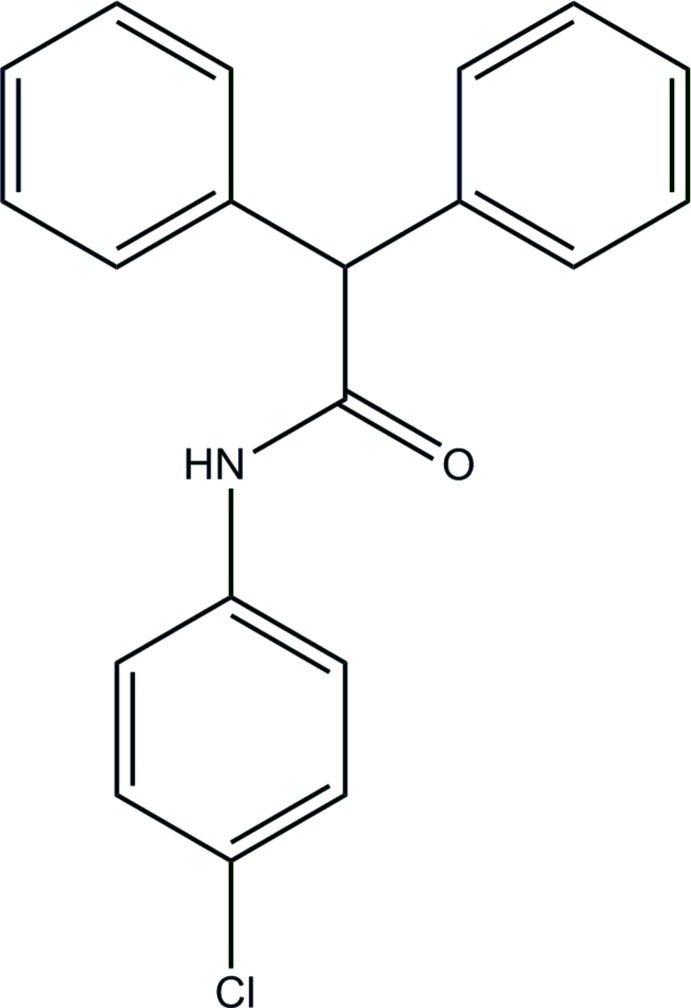


## Experimental

### 

#### Crystal data

C_20_H_16_ClNO
*M* *_r_* = 321.79
Monoclinic, 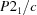

*a* = 10.2147 (2) Å
*b* = 17.8203 (4) Å
*c* = 9.5730 (2) Å
β = 114.019 (1)°
*V* = 1591.68 (6) Å^3^
*Z* = 4
Mo *K*α radiation
μ = 0.24 mm^−1^
*T* = 100 K
0.49 × 0.27 × 0.19 mm

#### Data collection

Bruker SMART APEXII CCD area-detector diffractometer
Absorption correction: multi-scan (*SADABS*; Bruker, 2009 ▶) *T*_min_ = 0.890, *T*_max_ = 0.954
14326 measured reflections
3639 independent reflections
3091 reflections with *I* > 2σ(*I*)
*R*_int_ = 0.028

#### Refinement

*R*[*F* ^2^ > 2σ(*F* ^2^)] = 0.044
*wR*(*F* ^2^) = 0.103
*S* = 1.09
3639 reflections
212 parameters
H atoms treated by a mixture of independent and constrained refinement
Δρ_max_ = 0.30 e Å^−3^
Δρ_min_ = −0.28 e Å^−3^

### 

Data collection: *APEX2* (Bruker, 2009 ▶); cell refinement: *SAINT* (Bruker, 2009 ▶); data reduction: *SAINT*; program(s) used to solve structure: *SHELXTL* (Sheldrick, 2008 ▶); program(s) used to refine structure: *SHELXTL*; molecular graphics: *SHELXTL*; software used to prepare material for publication: *SHELXTL* and *PLATON* (Spek, 2009 ▶).

## Supplementary Material

Crystal structure: contains datablock(s) global, I. DOI: 10.1107/S1600536812032965/rz2793sup1.cif

Structure factors: contains datablock(s) I. DOI: 10.1107/S1600536812032965/rz2793Isup2.hkl

Supplementary material file. DOI: 10.1107/S1600536812032965/rz2793Isup3.cml

Additional supplementary materials:  crystallographic information; 3D view; checkCIF report

## Figures and Tables

**Table 1:** Hydrogen-bond geometry (Å, °) *Cg*1 is the centroid of the C7–C12 ring.

*D*—H⋯*A*	*D*—H	H⋯*A*	*D*⋯*A*	*D*—H⋯*A*
N1—H1*N*1⋯O1^i^	0.84 (2)	2.07 (2)	2.8598 (19)	157 (2)
C1—H1*A*⋯O1	0.95	2.58	3.202 (3)	123
C4—H4*A*⋯*Cg*1^ii^	0.95	2.93	3.415 (2)	113

Symmetry codes: (i) 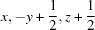; (ii) 
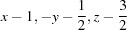
.

**Fig. 1.**
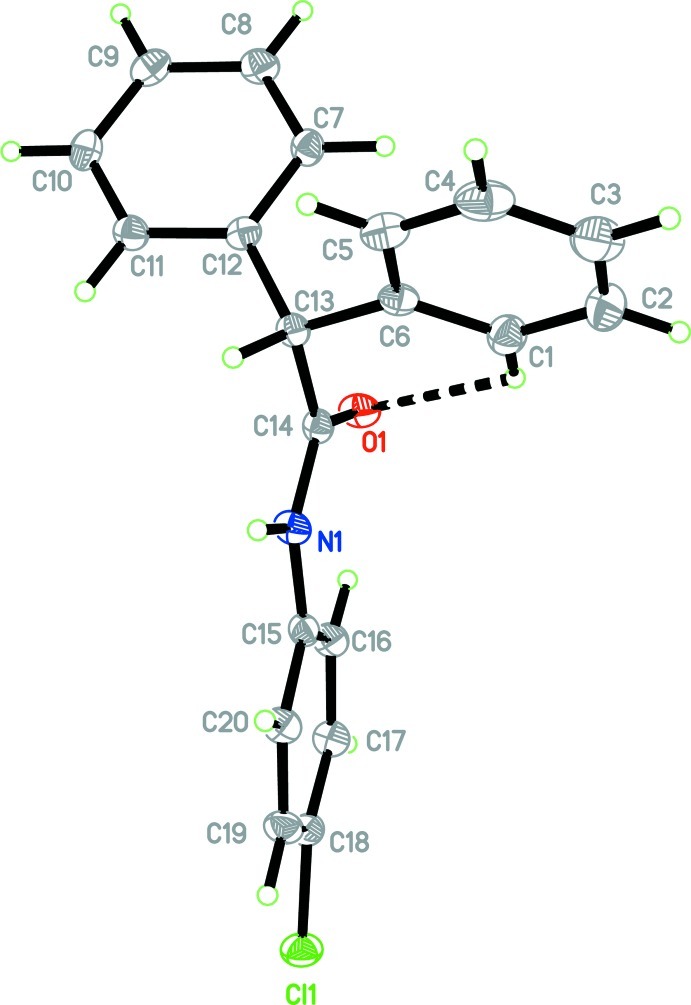
The molecular structure of the title compound, showing 50% probability displacement ellipsoids. The dashed line indicates the intramolecular hydrogen bond.

**Fig. 2.**
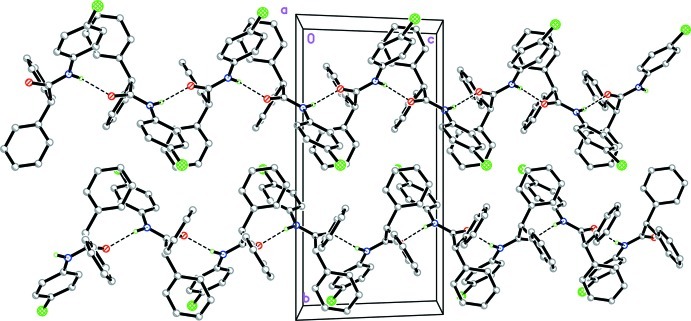
The crystal packing of the title compound, viewed along the a axis. H atoms not involved in the intermolecular interactions (dashed lines) have been omitted for clarity.

## supplementary crystallographic information

### Comment

In continuation of our work on the synthesis of amides (Fun *et al.*, 2012**a*,*b*,c*) we report herein the crystal structure of the title compound.

In the title compound, Fig. 1, an *S*(6) ring motif (Bernstein *et al.*, 1995) is formed *via* intramolecular C1—H1A···O1 hydrogen bond (Table 1). The chloro-substituted benzene ring (C15–C20) is almost perpendicular to the benzene rings (C1–C6, C7–C12) forming dihedral angles of 87.33 (9) and 88.69 (9)°, respectively. The dihedral angle formed by the benzene rings is 87.17 (9)°. Bond lengths (Allen *et al.*, 1987) and angles are within the normal ranges. In the crystal packing (Fig. 2), the molecules are linked into chains parallel the *c* axis by intermolecular N1—H1N1···O1 hydrogen bonds (Table 1). The crystal packing is further stabilized by weak C—H···π interactions (Table 1) involving the chloro-substituted ring.

### Experimental

Diphenylacetic acid (0.212 g, 1 mmol), 4-chloroaniline (0.127 g, 1 mmol) and 1-ethyl-3-(3-dimethylaminopropyl)-carbodiimide hydrochloride (1.0 g, 0.01 mol) were dissolved in dichloromethane (20 ml). The mixture was stirred in presence of triethylamine at 273 K for about 3 h. The contents were poured into 100 ml of ice-cold aqueous hydrochloric acid with stirring, which was extracted thrice with dichloromethane. The organic layer was washed with saturated NaHCO_3_ solution and brine solution, dried and concentrated under reduced pressure to give the title compound. Single crystals were grown from *N*,*N*-dimethylformamide by the slow evaporation method. M.p.: 463–465 K.

### Refinement

The N-bound H atom was located in a difference Fourier map and was refined freely [N–H = 0.84 (2) Å]. The remaining H atoms were located geometrically and refined using a riding model with *U*_iso_(H) = 1.2 *U*_eq_(C) [C–H = 0.95 Å].

### Crystal data

**Table:**

C_20_H_16_ClNO	*F*(000) = 672
*M**_r_* = 321.79	*D*_x_ = 1.343 Mg m^−^^3^
Monoclinic, *P*2_1_/*c*	Mo *K*α radiation, λ = 0.71073 Å
Hall symbol: -P 2ybc	Cell parameters from 8307 reflections
*a* = 10.2147 (2) Å	θ = 2.2–32.2°
*b* = 17.8203 (4) Å	µ = 0.24 mm^−^^1^
*c* = 9.5730 (2) Å	*T* = 100 K
β = 114.019 (1)°	Block, colourless
*V* = 1591.68 (6) Å^3^	0.49 × 0.27 × 0.19 mm
*Z* = 4	

### Data collection

**Table:**

Bruker SMART APEXII CCD area-detector diffractometer	3639 independent reflections
Radiation source: fine-focus sealed tube	3091 reflections with *I* > 2σ(*I*)
Graphite monochromator	*R*_int_ = 0.028
φ and ω scans	θ_max_ = 27.5°, θ_min_ = 2.2°
Absorption correction: multi-scan (*SADABS*; Bruker, 2009)	*h* = −13→13
*T*_min_ = 0.890, *T*_max_ = 0.954	*k* = −21→23
14326 measured reflections	*l* = −12→12

### Refinement

**Table:**

Refinement on *F*^2^	Primary atom site location: structure-invariant direct methods
Least-squares matrix: full	Secondary atom site location: difference Fourier map
*R*[*F*^2^ > 2σ(*F*^2^)] = 0.044	Hydrogen site location: inferred from neighbouring sites
*wR*(*F*^2^) = 0.103	H atoms treated by a mixture of independent and constrained refinement
*S* = 1.09	*w* = 1/[σ^2^(*F*_o_^2^) + (0.0323*P*)^2^ + 1.4338*P*] where *P* = (*F*_o_^2^ + 2*F*_c_^2^)/3
3639 reflections	(Δ/σ)_max_ = 0.001
212 parameters	Δρ_max_ = 0.30 e Å^−^^3^
0 restraints	Δρ_min_ = −0.28 e Å^−^^3^

### Special details

**Table:**

Experimental. The crystal was placed in the cold stream of an Oxford Cryosystems Cobra open-flow nitrogen cryostat (Cosier & Glazer, 1986) operating at 100.0 (1) K.
Geometry. All e.s.d.'s (except the e.s.d. in the dihedral angle between two l.s. planes) are estimated using the full covariance matrix. The cell e.s.d.'s are taken into account individually in the estimation of e.s.d.'s in distances, angles and torsion angles; correlations between e.s.d.'s in cell parameters are only used when they are defined by crystal symmetry. An approximate (isotropic) treatment of cell e.s.d.'s is used for estimating e.s.d.'s involving l.s. planes.
Refinement. Refinement of *F*^2^ against ALL reflections. The weighted *R*-factor *wR* and goodness of fit *S* are based on *F*^2^, conventional *R*-factors *R* are based on *F*, with *F* set to zero for negative *F*^2^. The threshold expression of *F*^2^ > σ(*F*^2^) is used only for calculating *R*-factors(gt) *etc*. and is not relevant to the choice of reflections for refinement. *R*-factors based on *F*^2^ are statistically about twice as large as those based on *F*, and *R*- factors based on ALL data will be even larger.

### Fractional atomic coordinates and isotropic or equivalent isotropic displacement parameters (Å^2^)

**Table:**

	*x*	*y*	*z*	*U*_iso_*/*U*_eq_	
Cl1	1.15388 (5)	0.49276 (3)	1.29695 (5)	0.02582 (13)	
O1	0.61313 (13)	0.26955 (7)	0.78711 (13)	0.0207 (3)	
N1	0.64271 (15)	0.29804 (8)	1.03055 (16)	0.0164 (3)	
H1N1	0.614 (2)	0.2863 (12)	1.098 (3)	0.027 (6)*	
C1	0.2968 (2)	0.32265 (11)	0.7336 (2)	0.0228 (4)	
H1A	0.3761	0.3418	0.7169	0.027*	
C2	0.1669 (2)	0.36130 (12)	0.6756 (2)	0.0315 (5)	
H2A	0.1578	0.4064	0.6194	0.038*	
C3	0.0508 (2)	0.33385 (12)	0.6999 (3)	0.0352 (5)	
H3A	−0.0377	0.3602	0.6609	0.042*	
C4	0.0645 (2)	0.26804 (12)	0.7810 (2)	0.0310 (5)	
H4A	−0.0149	0.2491	0.7977	0.037*	
C5	0.19353 (19)	0.22950 (11)	0.8381 (2)	0.0237 (4)	
H5A	0.2017	0.1842	0.8932	0.028*	
C6	0.31158 (18)	0.25666 (10)	0.81530 (19)	0.0179 (4)	
C7	0.37110 (18)	0.12810 (10)	0.6510 (2)	0.0182 (4)	
H7A	0.3131	0.1676	0.5905	0.022*	
C8	0.37547 (19)	0.05950 (10)	0.5837 (2)	0.0211 (4)	
H8A	0.3202	0.0525	0.4774	0.025*	
C9	0.45962 (19)	0.00140 (10)	0.6702 (2)	0.0213 (4)	
H9A	0.4622	−0.0453	0.6235	0.026*	
C10	0.54027 (19)	0.01183 (10)	0.8258 (2)	0.0206 (4)	
H10A	0.5984	−0.0277	0.8859	0.025*	
C11	0.53563 (18)	0.08021 (10)	0.8931 (2)	0.0184 (4)	
H11A	0.5907	0.0870	0.9996	0.022*	
C12	0.45162 (17)	0.13905 (10)	0.80713 (19)	0.0158 (3)	
C13	0.45186 (17)	0.21313 (10)	0.88570 (18)	0.0156 (3)	
H13A	0.4669	0.2009	0.9931	0.019*	
C14	0.57806 (17)	0.26222 (10)	0.89552 (19)	0.0155 (3)	
C15	0.76050 (17)	0.34768 (10)	1.08061 (19)	0.0160 (3)	
C16	0.84756 (18)	0.36106 (10)	1.0026 (2)	0.0192 (4)	
H16A	0.8252	0.3391	0.9052	0.023*	
C17	0.96742 (18)	0.40692 (10)	1.0687 (2)	0.0206 (4)	
H17A	1.0280	0.4158	1.0170	0.025*	
C18	0.99832 (18)	0.43946 (10)	1.2094 (2)	0.0199 (4)	
C19	0.90983 (19)	0.42853 (10)	1.2855 (2)	0.0209 (4)	
H19A	0.9307	0.4521	1.3812	0.025*	
C20	0.79109 (18)	0.38303 (10)	1.2206 (2)	0.0193 (4)	
H20A	0.7295	0.3757	1.2717	0.023*	

### Atomic displacement parameters (Å^2^)

**Table:**

	*U*^11^	*U*^22^	*U*^33^	*U*^12^	*U*^13^	*U*^23^
Cl1	0.0203 (2)	0.0244 (2)	0.0292 (3)	−0.00523 (17)	0.00640 (18)	−0.00464 (19)
O1	0.0268 (6)	0.0253 (7)	0.0134 (6)	−0.0066 (5)	0.0119 (5)	−0.0035 (5)
N1	0.0188 (7)	0.0221 (8)	0.0115 (7)	−0.0028 (6)	0.0095 (6)	−0.0009 (6)
C1	0.0239 (9)	0.0212 (9)	0.0236 (9)	0.0012 (7)	0.0100 (7)	−0.0002 (8)
C2	0.0332 (11)	0.0235 (10)	0.0336 (12)	0.0061 (8)	0.0093 (9)	0.0002 (9)
C3	0.0239 (10)	0.0314 (12)	0.0446 (13)	0.0058 (8)	0.0080 (9)	−0.0130 (10)
C4	0.0225 (9)	0.0328 (11)	0.0396 (12)	−0.0054 (8)	0.0148 (9)	−0.0146 (10)
C5	0.0244 (9)	0.0228 (10)	0.0262 (10)	−0.0050 (7)	0.0126 (8)	−0.0062 (8)
C6	0.0197 (8)	0.0191 (9)	0.0154 (8)	−0.0015 (7)	0.0078 (7)	−0.0064 (7)
C7	0.0217 (8)	0.0189 (9)	0.0155 (8)	0.0005 (7)	0.0090 (7)	0.0022 (7)
C8	0.0264 (9)	0.0232 (10)	0.0154 (8)	−0.0031 (7)	0.0101 (7)	−0.0035 (7)
C9	0.0268 (9)	0.0177 (9)	0.0248 (9)	−0.0017 (7)	0.0161 (8)	−0.0033 (7)
C10	0.0228 (9)	0.0185 (9)	0.0234 (9)	0.0021 (7)	0.0125 (7)	0.0046 (7)
C11	0.0202 (8)	0.0214 (9)	0.0146 (8)	−0.0019 (7)	0.0081 (7)	0.0004 (7)
C12	0.0178 (8)	0.0170 (8)	0.0163 (8)	−0.0030 (6)	0.0107 (7)	−0.0006 (7)
C13	0.0199 (8)	0.0185 (9)	0.0101 (8)	−0.0016 (7)	0.0079 (6)	0.0005 (7)
C14	0.0183 (8)	0.0161 (8)	0.0133 (8)	0.0016 (6)	0.0077 (6)	0.0010 (7)
C15	0.0169 (8)	0.0166 (8)	0.0136 (8)	0.0009 (6)	0.0053 (6)	0.0006 (7)
C16	0.0231 (9)	0.0217 (9)	0.0147 (8)	−0.0005 (7)	0.0095 (7)	−0.0012 (7)
C17	0.0202 (8)	0.0229 (9)	0.0205 (9)	−0.0012 (7)	0.0102 (7)	−0.0001 (8)
C18	0.0164 (8)	0.0168 (9)	0.0226 (9)	−0.0002 (7)	0.0041 (7)	−0.0003 (7)
C19	0.0230 (9)	0.0224 (9)	0.0160 (9)	0.0009 (7)	0.0068 (7)	−0.0054 (7)
C20	0.0206 (8)	0.0228 (9)	0.0168 (9)	0.0008 (7)	0.0099 (7)	−0.0023 (7)

### Geometric parameters (Å, º)

**Table:**

Cl1—C18	1.7451 (18)	C8—H8A	0.9500
O1—C14	1.2349 (19)	C9—C10	1.390 (3)
N1—C14	1.349 (2)	C9—H9A	0.9500
N1—C15	1.411 (2)	C10—C11	1.388 (3)
N1—H1N1	0.84 (2)	C10—H10A	0.9500
C1—C6	1.386 (3)	C11—C12	1.392 (2)
C1—C2	1.395 (3)	C11—H11A	0.9500
C1—H1A	0.9500	C12—C13	1.519 (2)
C2—C3	1.387 (3)	C13—C14	1.529 (2)
C2—H2A	0.9500	C13—H13A	1.0000
C3—C4	1.382 (3)	C15—C16	1.395 (2)
C3—H3A	0.9500	C15—C20	1.397 (2)
C4—C5	1.386 (3)	C16—C17	1.392 (2)
C4—H4A	0.9500	C16—H16A	0.9500
C5—C6	1.396 (2)	C17—C18	1.380 (3)
C5—H5A	0.9500	C17—H17A	0.9500
C6—C13	1.524 (2)	C18—C19	1.386 (2)
C7—C8	1.391 (3)	C19—C20	1.379 (2)
C7—C12	1.396 (2)	C19—H19A	0.9500
C7—H7A	0.9500	C20—H20A	0.9500
C8—C9	1.385 (3)		
			
C14—N1—C15	129.75 (14)	C10—C11—C12	121.05 (16)
C14—N1—H1N1	115.6 (15)	C10—C11—H11A	119.5
C15—N1—H1N1	114.4 (15)	C12—C11—H11A	119.5
C6—C1—C2	120.71 (18)	C11—C12—C7	118.73 (16)
C6—C1—H1A	119.6	C11—C12—C13	119.04 (15)
C2—C1—H1A	119.6	C7—C12—C13	122.23 (15)
C3—C2—C1	119.9 (2)	C12—C13—C6	114.31 (14)
C3—C2—H2A	120.0	C12—C13—C14	111.05 (13)
C1—C2—H2A	120.0	C6—C13—C14	110.69 (14)
C4—C3—C2	119.74 (19)	C12—C13—H13A	106.8
C4—C3—H3A	120.1	C6—C13—H13A	106.8
C2—C3—H3A	120.1	C14—C13—H13A	106.8
C3—C4—C5	120.29 (19)	O1—C14—N1	123.99 (16)
C3—C4—H4A	119.9	O1—C14—C13	122.31 (15)
C5—C4—H4A	119.9	N1—C14—C13	113.66 (14)
C4—C5—C6	120.65 (19)	C16—C15—C20	119.63 (16)
C4—C5—H5A	119.7	C16—C15—N1	124.52 (15)
C6—C5—H5A	119.7	C20—C15—N1	115.82 (15)
C1—C6—C5	118.67 (17)	C17—C16—C15	119.43 (16)
C1—C6—C13	123.22 (15)	C17—C16—H16A	120.3
C5—C6—C13	118.08 (16)	C15—C16—H16A	120.3
C8—C7—C12	120.19 (17)	C18—C17—C16	120.00 (16)
C8—C7—H7A	119.9	C18—C17—H17A	120.0
C12—C7—H7A	119.9	C16—C17—H17A	120.0
C9—C8—C7	120.61 (17)	C17—C18—C19	120.99 (16)
C9—C8—H8A	119.7	C17—C18—Cl1	119.93 (14)
C7—C8—H8A	119.7	C19—C18—Cl1	119.04 (14)
C8—C9—C10	119.58 (17)	C20—C19—C18	119.22 (16)
C8—C9—H9A	120.2	C20—C19—H19A	120.4
C10—C9—H9A	120.2	C18—C19—H19A	120.4
C11—C10—C9	119.83 (17)	C19—C20—C15	120.65 (16)
C11—C10—H10A	120.1	C19—C20—H20A	119.7
C9—C10—H10A	120.1	C15—C20—H20A	119.7
			
C6—C1—C2—C3	0.2 (3)	C5—C6—C13—C12	72.2 (2)
C1—C2—C3—C4	−0.3 (3)	C1—C6—C13—C14	16.8 (2)
C2—C3—C4—C5	0.1 (3)	C5—C6—C13—C14	−161.46 (15)
C3—C4—C5—C6	0.3 (3)	C15—N1—C14—O1	−1.8 (3)
C2—C1—C6—C5	0.2 (3)	C15—N1—C14—C13	−179.66 (16)
C2—C1—C6—C13	−178.09 (17)	C12—C13—C14—O1	43.3 (2)
C4—C5—C6—C1	−0.4 (3)	C6—C13—C14—O1	−84.76 (19)
C4—C5—C6—C13	177.91 (16)	C12—C13—C14—N1	−138.80 (15)
C12—C7—C8—C9	−0.1 (3)	C6—C13—C14—N1	93.11 (17)
C7—C8—C9—C10	0.1 (3)	C14—N1—C15—C16	−9.8 (3)
C8—C9—C10—C11	0.1 (3)	C14—N1—C15—C20	172.23 (17)
C9—C10—C11—C12	−0.3 (3)	C20—C15—C16—C17	2.8 (3)
C10—C11—C12—C7	0.2 (2)	N1—C15—C16—C17	−175.13 (16)
C10—C11—C12—C13	−179.13 (15)	C15—C16—C17—C18	−0.8 (3)
C8—C7—C12—C11	0.0 (2)	C16—C17—C18—C19	−1.4 (3)
C8—C7—C12—C13	179.30 (15)	C16—C17—C18—Cl1	176.51 (14)
C11—C12—C13—C6	−149.59 (15)	C17—C18—C19—C20	1.5 (3)
C7—C12—C13—C6	31.1 (2)	Cl1—C18—C19—C20	−176.42 (14)
C11—C12—C13—C14	84.30 (18)	C18—C19—C20—C15	0.6 (3)
C7—C12—C13—C14	−95.00 (18)	C16—C15—C20—C19	−2.7 (3)
C1—C6—C13—C12	−109.50 (18)	N1—C15—C20—C19	175.40 (16)

### Hydrogen-bond geometry (Å, º)

*Cg*1 is the centroid of the C7–C12 ring.

**Table:**

*D*—H···*A*	*D*—H	H···*A*	*D*···*A*	*D*—H···*A*
N1—H1*N*1···O1^i^	0.84 (2)	2.07 (2)	2.8598 (19)	157 (2)
C1—H1*A*···O1	0.95	2.58	3.202 (3)	123
C4—H4*A*···*Cg*1^ii^	0.95	2.93	3.415 (2)	113

Symmetry codes: (i) *x*, −*y*+1/2, *z*+1/2; (ii) *x*−1, −*y*−1/2, *z*−3/2.

## References

[bb1] Allen, F. H., Kennard, O., Watson, D. G., Brammer, L., Orpen, A. G. & Taylor, R. (1987). *J. Chem. Soc. Perkin Trans. 2*, pp. S1–19.

[bb2] Bernstein, J., Davis, R. E., Shimoni, L. & Chang, N.-L. (1995). *Angew. Chem. Int. Ed. Engl.* **34**, 1555–1573.

[bb3] Bruker (2009). *APEX2*, *SAINT* and *SADABS* Bruker AXS Inc., Madison, Wisconsin, USA.

[bb4] Cosier, J. & Glazer, A. M. (1986). *J. Appl. Cryst.* **19**, 105–107.

[bb5] Fun, H.-K., Chia, T. S., Nayak, P. S., Narayana, B. & Sarojini, B. K. (2012*a*). *Acta Cryst.* E**68**, o1316–o1317. 10.1107/S1600536812014079 PMC3344457 22590219

[bb6] Fun, H.-K., Chia, T. S., Nayak, P. S., Narayana, B. & Sarojini, B. K. (2012*b*). *Acta Cryst.* E**68**, o1287–o1288. 10.1107/S1600536812013451 PMC3344437 22590199

[bb7] Fun, H.-K., Ooi, C. W., Nayak, P. S., Narayana, B. & Sarojini, B. K. (2012*c*). *Acta Cryst.* E**68**, o1312–o1313. 10.1107/S1600536812013840 PMC3344455 22590217

[bb8] Sheldrick, G. M. (2008). *Acta Cryst.* A**64**, 112–122. 10.1107/S0108767307043930 18156677

[bb9] Spek, A. L. (2009). *Acta Cryst.* D**65**, 148–155. 10.1107/S090744490804362X PMC2631630 19171970

